# A Perspective on Robotic Assistance for Knee Arthroplasty

**DOI:** 10.1155/2013/970703

**Published:** 2013-04-30

**Authors:** Nathan A. Netravali, Feimo Shen, Youngbae Park, William L. Bargar

**Affiliations:** ^1^Curexo Technology Corporation, Fremont, CA 94539, USA; ^2^Department of Orthopaedics, University of California at Davis School of Medicine, Sutter General Hospital, Sacramento, CA 95816, USA

## Abstract

Knee arthroplasty is used to treat patients with degenerative joint disease of the knee to reduce pain and restore the function of the joint. Although patient outcomes are generally quite good, there are still a number of patients that are dissatisfied with their procedures. Aside from implant design which has largely become standard, surgical technique is one of the main factors that determine clinical results. Therefore, a lot of effort has gone into improving surgical technique including the use of computer-aided surgery. The latest generation of orthopedic surgical tools involves the use of robotics to enhance the surgeons' abilities to install implants more precisely and consistently. This review presents an evolution of robot-assisted surgical systems for knee replacement with an emphasis on the clinical results available in the literature. Ever since various robotic-assistance systems were developed and used clinically worldwide, studies have demonstrated that these systems are as safe as and more accurate than conventional methods of manual implantation. Robotic surgical assistance will likely result in improved surgical technique and improved clinical results.

## 1. Introduction

Reconstructive knee surgery, whether unicompartmental (UKA), multicompartmental (MCKA), or total knee arthroplasty (TKA), is commonly performed on patients with end-stage osteoarthritis of the knee. Currently, there are approximately 600,000 primary TKA procedures and 45,000 primary UKA procedures performed annually in the USA [1]. The number of procedures is growing rapidly with TKA growing at a rate of 9.4% per annum and UKA growing at a rate of 32.5% per annum in the United States [2]. The goal of a knee arthroplasty is to restore the knee joint to a functional and pain-free state. In terms of clinical outcomes, TKA is a successful procedure when looking at pain relief and restoration of patient mobility with 10–15 years implant survival rates of greater than 90% [3–5]. Similarly, UKA has a ten-year survival rate of over 90% [6].

However, the surgeries still need to improve in terms of patient satisfaction, especially in the case of younger patients. Patient satisfaction remains at only 82% to 89% after TKA [7–9]. Patients who received UKA are satisfied only 80–83% of the time [10]. Additionally, for younger patients, increased implant longevity and the ability to continue an active lifestyle are strongly desired. Both the survival rate of knee arthroplasty and patient satisfaction are dependent on multiple factors including patient selection, implant design, the preoperative condition of the joint, surgical technique, and rehabilitation.

When looking to improve implant survival and patient satisfaction, surgeons may choose from a variety of implants and different surgical techniques. The first factor is implant design. It includes component geometry, materials, and manufacturing processes and have changed since total knee arthroplasty first came about. However, patient satisfaction does not seem to have improved with these contemporary implants [7]. Although the majority of implants used today are generic, there are now custom implants based on a patient's individual anatomy (ConforMIS, Burlington, MA, USA). At this point, their use is too new to draw any conclusions regarding their effects on implant survival and patient satisfaction. The other factor is surgical technique which includes access to the joint, implant sizing, implant alignment, and positioning relative to anatomic features, implant fixation to the bone, soft tissue balancing, and wound closure [3, 11]. It has been suggested that errors in surgical technique may be the most common reason for failure of TKAs [3, 12, 13]. Thus, many recent developments in knee reconstructive surgery have focused on improvements in surgical technique. 

The traditional surgical technique involves bone cuts and soft tissue balancing. Bone cuts are typically performed with reference to anatomical landmarks and available implant geometry. Correct implant sizing is achieved when the native dimensions of the knee are reproduced as closely as possible by the implant. Conventional TKA instruments typically use intraoperative sizing guides to help the surgeon determine the appropriate implant size. In terms of implant to bone fixation, most knee replacement implants are attached to the host bone using bone cement (PMMA). In the alternative, cementless fixation, the implants generally have porous regions adjacent to the bone designed to allow for bony ingrowth. While bone cement provides good initial fixation even with poor quality bone, cementless fixation provides direct bone-to-metal attachment, which reduces migration after an initial period and thus may lead to a potentially longer implant life [14]. To achieve reliable cementless fixation, precision bone cuts must be made so that the implants achieve stable initial fixation with limited gaps. However, with conventional instruments, the bone cuts are made using bone-attached cutting guides and an oscillating saw. As demonstrated by Plaskos et al. [15], these cutting guides combined with an oscillating saw resulted in errors in cuts ranging from 0.6° to 1.1° in varus-valgus and 1.8° in flexion-extension. These cutting errors can result in gaps that delay bone ingrowth into the implant [16] or may require bone cement to ensure initial stability.

The postoperative alignment of the knee has a large effect on the load transferred through the implant. To spread the load evenly, manufacturers have traditionally recommended positioning the knee implants (totals or partials) such that the “ideal” mechanical axis of the leg is restored. This mechanical axis is defined as a straight line passing from the center of the femoral head to the center of the talus [17, 18]. In addition, the implants should be positioned such that the anatomic joint line is preserved or restored and minimal bone is removed. Although not all studies agree [19, 20], many studies have shown that restoring a neutral postoperative mechanical axis, defined by the center of the hip, center of the knee, and center of the ankle within ±3° of the mechanical axis, may result in improved postoperative pain, biomechanics, function, and an increased implant longevity [17, 21–23].

Traditional planning for implant positioning and alignment is done using acetate implant overlays on appropriately magnified radiographs of the knee [24]. During the actual surgery, mechanical alignment jigs are used to assist in making the bone cuts. These jigs reference the long axis of the bone either by estimating it externally or internally entering the intramedullary canal. Cutting guides are attached to the bones and a hand-held oscillating saw is used to perform the bony cuts.

With regards to soft tissue balancing, there are two main techniques employed by surgeons. The first is called the “gap balancing” technique. This method determines the rotational and AP position of the femoral component intraoperatively in an attempt to achieve a rectangular flexion gap equal to or close to the extension gap. This will theoretically achieve ligament balance, but may result in a nonanatomic alignment of the femoral component. The second method is called the “measured resection” technique. The measured resection technique relies on the intraoperatively determined location of the transepicondylar axis (TEA). The TEA has been shown [25, 26] to be the best indicator of a patient's true anatomic flexion axis. However, locating the TEA intraoperatively can be difficult due to osteophytes and problems that may arise with adequate exposure. Thus, several other alignment measures are often used instead of the TEA, such as Whiteside's line. Although Whiteside's line is likely easier to locate, it is also prone to error. As such, many surgeons will simply place the femoral component in a fixed position of external rotation (typically 3°) relative to the posterior condylar axis as an estimation of the TEA. Although this position is easy to find repeatedly, its relationship to the TEA is variable and can result in unequal ligament balance [27, 28].

Implant manufacturers have developed complex manual instrumentation to address each of the above factors and help the surgeon place the implants where they planned. Numerous peer-reviewed published papers have identified knee alignment as the most important factor in achieving good long-term clinical results [17, 21, 23, 29–42]. In addition to manual instruments, computer navigation and robotic systems have been developed to increase the accuracy of implant placement and knee alignment and reduce outliers with the overall goal of improved long-term clinical results. 

## 2. Computer Assisted and Robotic-Assistance Surgery Systems

Computer assisted surgical systems include a variety of methods to address many of the challenges associated with knee arthroplasty. Surgical navigation systems typically provide the surgeon with information including bone orientations and limb alignments through a display. Additionally, patient-specific instrumentation and implants are now being used [53, 54]. These systems typically require computer-assisted planning and design of the instrumentation. They can assist the surgeon in creating a surgical plan or guiding surgical tools. These passive systems may be classified outside of the robotic realm. 

Robotic assistive systems are robotic devices that perform specific tasks according to preoperative data. These systems can be classified into three main categories: passive systems, semiactive robotic systems, and active robotic systems [55]. Passive systems perform part of the surgical procedure under continuous and direct control of the surgeon. An example of a passive system is one in which a robot holds a guide or jig in a predetermined location and the surgeon uses manual tools to prepare the bony surfaces. A semiactive robotic system is a tactile feedback system that augments the surgeon's ability to control the tool typically by restricting the cut volume by defining constraints of the cut motion in space; however, it still requires the surgeon to manipulate the cutter. Finally, an active robotic system performs a surgical task without direct intervention of the surgeon such as allowing the robotic arm to cut the bone without direct manipulation of the cutter by the surgeon.

Although navigation systems have been shown to reduce the number of mechanical axis alignment outliers [56], the actual cutting of bone relies on manual tools which limit the accuracy of the cuts [29]. For this reason, surgeons and engineers have worked to integrate robotically controlled surgical instruments into joint replacement surgery [40]. In addition to the computer-controlled cutting instrument, robotic systems use CT-based three-dimensional (3D) visualization and templating to plan the cuts. This allows easier preoperative identification of anatomical landmarks such as the TEA. Most robotic systems consist of very similar components. The steps to a robotically-assisted surgery typically involve (1) creating a patient specific model and interventional plan; (2) intraoperatively registering the model and plan to the patient's anatomy; and (3) using robotic-assistance to make bone cuts and carry out the preoperative plan on the patient. 

Matsen et al. [57] were the first to describe a robotic system for knee arthroplasty. Their passive system was based on a robot positioning saw and drill guides with respect to the bony geometry. Kienzle et al. [58] developed another passive system that used a preoperative CT scan and a pin-based registration technique. The preoperative CT allowed the surgeon to plan and accurately execute implant placement based on 3D reconstructions of the bones. van Ham et al. [59] presented a semiactive system in which the robot constrains the motion of the cutting tool as it is guided by the surgeon. This system used an intraoperative registration method using an intramedullary rod. Martelli et al. [60] presented a passive robotic system for use in TKA based on preoperative CT. Intraoperative registration was performed using a surface-matching technique based on the surface models created from the CT scans. Glozman et al. [61], La Palombara et al. [62] and Fadda et al. [63] used similar surface matching techniques to register bones without fiducial markers. These registration methods were then combined with active or semiactive robots that provided precision bone milling according to the preoperative plan.

In addition to these larger robots, there has been development of miniature bone-mounted robots. For example, PiGalileo (Plus Orthopedics AG, Smith & Nephew, Switzerland) is a passive system that uses a hybrid navigated robotic device that clamps on to the mediolateral aspects of the distal femoral shaft. The MBARS (Mini Bone-Attached Robotic System) was an active system developed for patellofemoral joint replacement procedures [64]. Plaskos et al. presented Praxiteles in 2005, as a passive system that is a miniature bone-mounted robot for total knee arthroplasty. Song et al. [65] have developed an active system consisting of a hybrid bone-attached robot for joint arthroplasty (HyBAR) that uses hinged prismatic joints to provide a structurally rigid robot for minimally invasive joint arthroplasty.

Although many of these systems have been developed and prototyped, only a handful have been used successfully in clinical settings throughout the world. These include the ROBODOC System (Curexo Technology Corporation, Fremont, CA), the CASPAR system (URS Ortho Rastatt, Germany), the Robotic Arm Interactive Orthopedic System (RIO; MAKO Surgical Corporation, Fort Lauderdale, FL, USA), and the Stanmore Sculptor Robotic Guidance Arm (RGA) System (Stanmore Implants, Elstree, UK), formerly known as the Acrobot System. MAKO's RIO and the Stanmore Sculptor RGA System are semiactive systems, whereas the CASPAR and ROBODOC systems are active robotic systems.

## 3. Clinical Results

A summary of published clinical studies in which robotic-assistance systems are used for TKA is presented in Table 1. The studies and their primary findings are described in the sections below for each individual system. 

### 3.1. CASPAR

A study using CASPAR for TKA was performed by Siebert et al. in [43]. Seventy CASPAR-assisted surgeries were compared to 52 control surgeries performed in Kassel, Germany. Postoperative standing long-leg radiographs showed that the robot group had a higher accuracy in achieving the planned femoral-tibial alignment with an average error of 0.8° (range 0–3°) compared to the control group's average error of 2.6° (range 0–7°). Another study followed 25 TKA cases that were consecutively performed using the CASPAR system [44]. Postoperative followup ranged from 5.1 to 5.8 years. The results demonstrated that all angular measurements for the tibial and femoral components in this study were within 1° of the target as defined in the preoperative plan. Operating time for these first 70 cases averaged 135 minutes but towards the end of the study achieved a steady state of approximately 90 minutes, which is approximately equal to the control group. No major adverse events related to the CASPAR system were found, but one minor complication was recorded. One TKA in one patient was successfully converted to a manual technique after a femoral milling could not be completed due to a defective registration marker. Additionally, three patients had superficial skin irritations at the pin sites that were resolved using conservative treatment.

### 3.2. Stanmore Sculptor RGA

The Stanmore Sculptor RGA system, previously known as the Acrobot System, was utilized in a randomized study performing unicompartmental knee arthroplasty (UKA) [45, 66]. This study included 13 patients undergoing Acrobot-assisted surgery and 15 patients undergoing UKA using conventional techniques. Postoperative CT scans showed that the femoral-tibial alignment for all 13 patients in the Acrobot-assisted group was less than 2° from the goal, whereas only 6 of the 15 patients in the conventional group had femoral-tibial alignments in this range. The functionality scores (American Knee Society) measured at 6 months postoperatively were also better for the patients operated using Acrobot. The operative time was typically about 10 minutes longer than conventional cases.

### 3.3. MAKO RIO

The MAKO Tactile Guidance System was used in a pilot study for UKA at Pennsylvania Hospital, Philadelphia, PA, USA using robot assistance from MAKO [46]. The study included 31 consecutive patients who underwent UKA using robotic arm assistance and 27 consecutive patients who underwent UKA performed with conventional manual instrumentation. Postoperative radiographs showed that the root mean square (RMS) error of the posterior tibial slope was 3.1° using manual techniques and 1.9° using robotic arm assistance. The average error of tibial alignment in the coronal plane was 2.7° ± 2.1° (mean  ±  standard deviation (SD)) using the conventional instruments compared with 0.2°  ± 1.8° (mean ± SD) using robotic arm assistance. Varus-valgus RMS error was 3.4° manually compared with 1.8° robotically.

 Another feasibility study was performed by Pearle et al. [67] in which 10 subjects needing a UKA were included. The results of this study showed that all of the patients had tibiofemoral angles in the coronal plane that were within 1° of what had been planned. There were no complications with the system and the wounds healed successfully.

 A third feasibility study was reported by Sinha [48] involving their first 20 cases. All of the 20 cases were successfully completed as planned, and the results showed a good ability to recreate individual patient anatomy. Prior to surgery, 62.5% of the knees were in varus and 37.5% were in valgus. The surgeries were planned to maintain this alignment, and, after surgery, all of the knees succeeded in matching their preoperative alignment. There were no outliers in terms of flexion. With respect to the tibiae, they were all varus prior to surgery and this was maintained as preoperatively planned. The mean tibial slope prior to surgery was 5.00 ± 2.37° (mean ± SD) with 25% outliers (defined as <0° or >7°), and after surgery the mean slope was 4.29 ± 3.24° (mean ± SD) with 19% outliers. Sinha reported no failures using the system in the first 20 patients, but reported one failure of tibial registration in the next 17 patients. This patient was successful converted to a manual technique.

 Coon et al. [49] compared 45 minimally invasive UKAs, performed using manual instrumentation, with 36 UKAs performed with RIO. They compared the Knee Society Scores (KSS) between the two groups postoperatively. There was no significant difference in terms of average KSS, change in KSS, or Marmor ratings between the two groups. This suggested that the RIO provides comparable clinical results to manual techniques for UKA. 

 Coon et al. [50] also compared a group of 44 UKA's performed using manual instrumentation with 33 UKA's using the RIO. The goal using both techniques was to match the natural tibial posterior slope, and the results showed that the RMS error using the manual technique was 3.5° and the error using the robotic system was 1.4°. Additionally, the variance using the manual instruments was 2.8 times greater than using the RIO. In the coronal plane, the manual instruments resulted in an average error of 3.3 ± 1.8° (SD) of varus compared to 0.1 ± 2.4° (SD) for the robotic system. Thus, the RIO resulted in improved accuracy in terms of implant placement during UKA when compared to manual instrumentation. 

### 3.4. ROBODOC

The ROBODOC System has been used clinically for TKA since 2000. The first 100 ROBODOC TKA procedures were performed by Professor Martin Börner at the Trauma Clinical of Trade Associations (BGU) in Frankfurt, Germany [30]. All of the patients received the Duracon Total Knee (DePuy Orthopedics Inc., Warsaw, IN, USA).

In this study, the results showed that the ROBODOC system made cuts that were good enough to allow cementless implantation for both the tibia and femur in 76 of the first 100 patients. Sixteen of the remaining cases needed cement for the tibial component and 8 cases needed cement for both components due to poor bone quality. In 97% of the cases, the alignment of the knee was restored to the planned ideal mechanical axis (0° error). The remaining three cases resulted in knee alignment being restored to within 1° of the ideal mechanical axis. The operating time decreased from 130 minutes for the first case to a typical time between 90 and 100 minutes by the end of the study. Of the first 100 cases, five were successfully converted to a manual procedure due to technical issues with the ROBODOC system.

Another study was recently published by Song et al. [51] looking at a direct comparison between a ROBODOC-assisted TKA and a manual TKA in the same subject using a prospective randomized study. Thirty patients underwent simultaneous bilateral TKA with a ROBODOC-assisted procedure in one knee and a manual procedure in the contralateral knee. The alignment of the knee and the individual components were determined postoperatively along with clinical follow-up scores including the HSS and WOMAC scores. The results showed significantly fewer outliers in terms of alignment errors and nearly equivalent clinical outcome results for both HSS and WOMAC scores. The postoperative mechanical axis was improved to 0.2°  ± 1.6° (mean ± SD) in the ROBODOC group and only 1.2°  ± 2.1° (mean ± SD) in the manual group. Furthermore, the ROBODOC group had no outliers in mechanical axis, defined as an error ≥± 3°, while the manual group had seven outliers. However, the ROBODOC-assisted surgeries took, on average, 25 minutes longer than the manual cases, but resulted in significantly less postoperative bleeding. There were no major adverse events related to the use of the robotic system reported.

Song et al. [52] also recently published another study comparing ROBODOC-assisted and manual TKAs. This study looked at 100 total subjects that were randomly divided into 50 receiving ROBODOC-assisted TKA and 50 receiving manual TKA. Once again, the main goal was to improve the mechanical axis alignment to neutral (0°). The results showed that the postoperative mechanical axis was improved to 0.5°  ± 1.4° (mean ± SD) in the ROBODOC-assisted group and 1.2°  ± 2.9° (mean ± SD) in the manual group. The ROBODOC group had significantly fewer outliers (0), once again defined as error ≥±3°, compared to the manual group (12). The operative time was once again of an average of 25 minutes longer in the ROBODOC cases, but they once again resulted in significantly less blood loss. The clinical results (range of motion, HSS scores, and WOMAC scores) showed no differences between the two groups. Additionally, this study compared the ability to balance the flexion and extension gaps after the bony cuts and soft tissue balancing were completed. The ROBODOC group resulted in only three outliers (defined as a difference in flexion and extension gap outside of 2 ± 2 mm (mean ± SD)) which were significantly fewer than the ten outliers found in the manual group. Finally, the PCL tension was measured intraoperatively. The ROBODOC group resulted in 96% of the knees having excellent tension and 4% having poor tension, while the manual group only had 76% of the knees with excellent tension and the remaining 24% with poor tension. This difference between groups was statistically significant. The ROBODOC group experienced six local and five systemic complications compared to the manual group which experienced three local and eight systemic complications. These complication rates were not statistically different.

## 4. The Future

Knee arthroplasty is widely considered a successful procedure in terms of relieving pain and improving function [3]. Yet, recent studies [7, 68, 69] have demonstrated that patient satisfaction is still less than optimal. Although the primary aim of knee replacement is relief of pain, once this outcome measure is achieved, patients' priorities may change and they may expect their procedure to enable them to return to original functional status, especially in younger patients [69]. Thus, the ability to accurately preoperatively plan to restore alignment or proper joint kinematics of the knee and then execute the plan is important in increasing patient's functionality, increasing the longevity of the implant, and reducing pain [17, 22, 23, 29–41, 51, 52]. Computer-assisted navigation surgery is a valuable technological development in orthopedics; however, robot-assisted surgery can achieve an improved level of accuracy and precision that is not possible with navigation alone. The use of robotic technology takes implant placement accuracy with navigation one level further by using information-rich 3D data during preoperative planning in combination with robot-controlled mechanical precision during implementation. This combination allows the surgeon to begin with a better plan for implant positioning and reduces the inevitable margin of error associated with manual preparation [15, 29] of the bone surfaces by the surgeon with or without navigation. The clinical results presented above show that robot-assisted orthopedic surgeries can already safely and effectively enhance the accuracy and precision of knee replacement without any major adverse events reported in any of the studies.

The potential benefit of precise implantation may be clouded by a lack of sensitivity in outcome measurement techniques. Clinical outcomes after knee arthroplasty are typically measured using objective functional outcome scoring systems that depend on postoperative pain and function. The most widely used scales include The Hospital for Special Surgery score (HSS, [70]), the Knee Society score (KS, [71]), the Western Ontario and McMaster Universities Osteoarthritis Index (WOMAC, [72]), the Oxford Knee Score [73], and the more generic Short Form-36 (SF-36, [74, 75]). These functional outcome scoring systems are completed either by the patients (WOMAC, SF-36, and Oxford Knee Score) or the clinicians (HSS, KS) and typically have a limited number of levels differentiating between the extremes of pain and functionality. Depending on the specific scoring system, there are only 4-5 gradations between these two extremes, and thus an individual with full functionality and another individual with approximately 90% functionality while walking will be counted in the same group. This may explain the differences found between patient and surgeon satisfaction when considering outcomes [7, 68, 69]. The criteria for a successful procedure may differ between patient and surgeon and thus outcomes may be exaggerated when reported by the surgeon based on outcome scores [76]. Taking this into account, Noble et al. [77] have recently introduced an updated Knee Society Scoring System that accurately addresses patient satisfaction, patient expectations, and patient symptoms while participating in a broad range of activities of daily living and activities important to each patient.

Furthermore, there is some debate as to what the ideal target for coronal plane alignment [19, 20]. Although navigation has been shown to reduce the number of outliers when looking at alignment, it has not been shown to improve short-term results, suggesting that coronal plane alignment may not be related to postoperative outcome [47]. In any case, the clinical studies reviewed in this paper demonstrate that robotic systems allow surgeons to better achieve their goals. If and when these alignment goals change, robotic systems are poised to better help surgeons achieve them in the future.

Despite all the benefits, there is still room for improvement with these robotic systems. The CASPAR system is no longer manufactured or being used clinically, but the Sculptor RGA, MAKO RIO, and ROBODOC systems are being used worldwide. Perhaps the biggest disadvantage of using a robot-assisted system for total knee replacement is the increase in operative time. The ROBODOC system results in a longer operative time compared to manual cases [51, 52]. The MAKO RIO and Sculptor RGA also require an increased operative time due to navigation and burring [47, 67, 78].

It should be noted that not all of these robotic systems have been used clinically without technical problems. A study by Chun et al. [79] examined the potential causes that can lead to aborting a ROBODOC arthroplasty procedure. Of 100 consecutively planned ROBODOC-assisted arthroplasties, the surgeons aborted 22 cases for a variety of reasons including registration failure, robot workspace issues, and potential damage to the patellar tendon. Of the aborted cases, only one resulted in complications with partial damage to the patellar tendon. Similarly, these issues exist for other robotic systems as evidenced by Sinha [48] who reported a failed tibial registration in the first 37 cases. 

Additionally, the cost of these systems is substantial in some cases. While the Stanmore Sculptor RGA is currently being offered at no cost to the surgeon, the initial capital equipment cost for robotic systems can be up to $800,000 [1]. Furthermore, the per case disposable costs associated with these procedures are higher than those associated with conventional procedures. Some of these extra costs can be mitigated by the fact that a reduced inventory for implants is needed for each procedure since the exact implant size is known prior to beginning the surgery based on the preoperative plan. Yet, the overall cost of implementing these systems typically remains increased. 

Robotic systems may affect implant design in the future. For example, patient-specific implants and instrumentation are currently available and are designed based on the patient's individual anatomy. However, the bone-implant interface for these systems is still designed to be compatible with traditional manual tools, such as oscillating saws and reamers. On the other hand, robotic systems, especially active systems, are capable of providing a precise freeform surface or an undercut shape that is virtually impossible with manual tools. With this ability, the implants can be designed with different surgical approaches or different fixation methods that may provide better initial stability using cementless fixation. 

The development of less invasive methods using robotic systems could result in faster recovery times and enhanced postoperative patient functionality. Robotic systems have the ability to work through smaller incisions than traditional instruments due to the ability to preplan the cutting path in an active system or restrict the movement of the cutter in a semiactive system. This can protect the soft tissues around the joint which can help with postoperative recovery and patient satisfaction.

Robotic assistance can clearly improve the accuracy of implant placement and fit in knee arthroplasty. These benefits may lead to robotic assistance becoming the gold standard for not only knee arthroplasty, but all joint arthroplasty because the principle of resecting bones, based on a preoperative plan is the same regardless of the bony geometry. Robotic-assisted orthopedic surgery systems are currently capable of improving a surgeon's ability to implement his/her preoperative plan. Although the clinical outcomes reported thus far for TKA using robotic systems are similar to those performed manually, the development of better more sensitive outcome measures such as the new Knee Society Scoring System [77] or gait analysis may be able to demonstrate benefits not apparent using current outcome measures. In the future, surgeons may be able to restore knee joints through even smaller incisions exactly as planned as robotic assistance becomes the standard in joint arthroplasty.

## Figures and Tables

**Table 1:** Clinical studies using robotic-assistance for unicompartmental or total knee arthroplasty.

Study	System	Procedure	# Robotic cases	# Conventional cases
Siebert et al. [43]	CASPAR	TKA	70	52
Bellemans et al. [44]	CASPAR	TKA	25	N/A
Cobb et al. [45]	Sculptor RGA	UKA	13	15
Lonner et al. [46]	MAKO	UKA	31	27
Pearle et al. [67]	MAKO	UKA	10	N/A
Sinha [48]	MAKO	UKA	20	N/A
Coon et al. [49]	MAKO	UKA	36	45
Coon et al. [50]	MAKO	UKA	33	44
Börner et al. [30]	ROBODOC	TKA	100	N/A
Song et al. [51]	ROBODOC	TKA	30	30
Song et al. [52]	ROBODOC	TKA	50	50
